# Supplementary material for: Nepal Pioneer Worksite Intervention Study to lower cardio-metabolic risk factors: design and protocol
Source: BMC Cardiovasc Disord. 2019 Feb 28;19:48. doi: 10.1186/s12872-019-1025-3 (PMC6393979; doi:10.1186/s12872-019-1025-3)
Supplement: Supplementary file 1 — Checklist to monitor cafeteria intervention in the Nepal Pioneer Worksite Intervention Study. (DOCX 17 kb) [file 12872_2019_1025_MOESM1_ESM.docx]

Additional file 1: Checklist to monitor cafeteria intervention in the Nepal Pioneer Worksite Intervention Study

Date

Name of the Cafeteria

Observer’s Name

Checklist (please check ‘yes’ or ‘no’)

| **SN** | **Observation (Mention Yes or No after observation in the cafeteria)** | **Yes** | **No** | **Remarks** |
| --- | --- | --- | --- | --- |
|  | 9 AM |  |  |  |
| 1 | Fruits (apple/banana) is available for breakfast |  |  |  |
| 2 | Whole grains (Oats, whole wheat flakes, whole wheat roti) are available for breakfast |  |  |  |
| 3 | Water dispenser is filled with water |  |  |  |
| 4 | Whole grain bread options for bread omelet, fried bread, jam bread |  |  |  |
| 5 | Separate sugar offered for milk, tea and coffee |  |  |  |
| 6 | Unhealthy foods (biscuits/ puff/ donuts) are available |  |  |  |
|  | 1 PM |  |  |  |
| 7 | Brown rice is mixed with white rice (1:1 ratio) |  |  |  |
| 8 | Whole wheat roti is available |  |  |  |
| 9 | Salads (cucumber, radish) are available |  |  |  |
| 10 | Salads (cucumber, radish) are placed before rice |  |  |  |
| 11 | Chicken curry, chicken chilli, chicken fried do not have skin and fats |  |  |  |
| 12 | Water dispenser is filled with water |  |  |  |
| 13 | Chicken momo, veg momo have whole wheat flour mixed with all-purpose flour |  |  |  |
|  | 4 PM |  |  |  |
| 14 | Whole grain (Popcorn) is available |  |  |  |
| 15 | Fruits (Banana, Apple, Oranges, Pomegranate, Papaya) are available |  |  |  |
| 16 | Water dispenser is filled with water |  |  |  |
| 17 | Beaten rice , thukpa and chowmein have 50% vegetables or lentils |  |  |  |
| 18 | Healthy plate poster is intact |  |  |  |
| 19 | Whole grain poster is intact |  |  |  |
| 20 | Soda (Coke, Fanta, Sprite, Mt Dew) is available |  |  |  |
